# Supplementary material for: Are carbon emissions trading and green financial instruments synergistic? -Comprehensive quantitative research based on content analysis
Source: PLoS One. 2024 Mar 7;19(3):e0298601. doi: 10.1371/journal.pone.0298601 (PMC10919601; doi:10.1371/journal.pone.0298601)
Supplement: S1 Data — (ZIP) [file pone.0298601.s001.zip › Data/地级市Z_F工作报告.zip!/║■▒▒/╛ú╓▌╩╨/└┤╫╘╣1⁄2╓┌║┼í╛┬φ┐╦╩2╛▌═°í┐_2010.docx]

现在，我代表市人民政府，向大会作工作报告，请予审议，并请市政协委员和列席人员提出意见。

2009年，是形势严峻、发展困难的一年，也是克难奋进、成效突出的一年。在省委、省政府和市委的正确领导下，在市人大、市政协的监督支持下，市政府深入开展学习实践科学发展观活动，认真贯彻落实中央应对国际金融危机的政策措施，团结带领广大干部群众，开拓创新，扎实工作，全市经济社会保持发展加快、质量提高、后劲增强、民生改善的良好态势。除外需不足带来出口下降外，市三届人大五次会议确定的目标任务圆满完成。实现生产总值707.8亿元、增长13.5%，全社会固定资产投资435.2亿元、增长49.3%，社会消费品零售总额445.4亿元、增长20.5%，财政收入50.2亿元、增长19.6%，万元生产总值综合能耗、化学需氧量和二氧化硫排放分别下降7.4%、6.2%和2.7%，城镇居民人均可支配收入13304元、增长9.1%，农民人均纯收入5464元、增长11.8%，城镇登记失业率为4.3%，人口自然增长率为3.4‰，居民消费价格指数下降1.2个百分点。

围绕扩大投资保增长，扎实开展“项目建设年”活动，大力推进项目策划、招商和建设，固定资产投资持续大幅增长，连续四年超全省平均水平，提前完成“十一五”投资千亿元目标。争取中央拉动内需投资15.1亿元，完成城镇以上固定资产投资384.8亿元、增长50.7%。招商引资成效明显，到位资金205.5亿元、增长23.8%，美的电器、三安弘晟光电、雨润食品、草本工房等一批重大项目相继落户建设。交通完成投资28亿元，东荆河大桥、荆州至江陵一级公路建成通车，盐卡港二期和随岳南高速主体工程完工，荆岳长江大桥即将合拢，汉宜铁路荆州段路基工程完成60%以上，荆岳铁路、洪监高速、江南高速等项目前期工作有序开展。水利完成投资12.8亿元，荆南四河堤防加固工程建设投入不断增加，四湖流域综合治理纳入国家规划，新滩口、东港等8处泵站更新改造基本完工，洈水、沙港等14座水库除险加固工程完成年度任务。电力、电信分别完成投资32.9亿元和 3.1亿元，供电通信保障能力进一步提高。

把推进新农村建设作为“三农”工作的重中之重，带动了农村新变化、农业新发展。仙洪试验区规模扩大，7个乡镇新纳入试验区范围，在交通、水利、血防、村庄整治及高产农田、特色板块建设等方面取得明显成效，乡村面貌发生深刻变化，成为全省新农村建设的亮点。积极推广试验区经验，扩大示范效应，8个省级试点镇完成规划编制，基础设施建设、环境整治等工程有序推进。全市建成通村公路2423公里、户用沼气池4.4万口、改厕11.5万座，新增农村安全饮水人口30万人。市政府分别与省农业厅、省林业局等单位签订战略合作协议，共建现代农业、水产产业示范园区、粮食加工园区，合作推进现代林业和生态文明建设，投入农业综合开发资金1.18亿元。扎实推进农业结构调整和产业化经营，主要农产品全面增产，粮、油、生猪、家禽、水产品等产量创历史新高，监利县荣获全国粮食生产先进县标兵称号。粮食总产74.1亿斤、增5.2亿斤，棉花263.2万担、减36.8万担，油菜达到1080.2万担、增111.6万担，生猪出栏397万头、增39万头，家禽出笼5947万只、增1184万只，水产放养面积211.9万亩、增23.9万亩，产量100万吨、增15.6万吨，完成植树造林27.3万亩。监利福娃、石首林业、洪湖水产等加工园区规模扩大，双港养殖加工、温氏畜禽、宏凯油脂等产业化企业发展加快，市级以上龙头企业达到187家，新增农民专业合作社305家、标志性农产品18个，农机综合作业率达到55%、提高3个百分点。认真落实强农惠农政策，发放农业补贴资金7.24亿元，农民负担总额1.3亿元、减少836.1万元。加大扶贫开发力度，老区、贫困地区投入扶贫资金7356万元，孟溪大垸整合资金投入1.96亿元。

把创建省级卫生城市作为提升城市形象品位的重要载体，推动了人居环境进一步改善。实施中心城区城市管理新体制，实行领导包路责任制，开展社区环境卫生、公共卫生“五小行业”、集贸市场、交通秩序、建筑工地等八大专项整治，动员机关企事业单位、街道社区增加投入、强化管理，推广“彭国珍服务队”和“雷锋互助社”等社区志愿服务模式，依法拆违30多万平方米，城市管理中多年积淀、居民反映强烈的一批老大难问题得到有效解决，实现了创建省级卫生城市目标。城市总规修编通过部际联席会议评审，分区规划和控制性详规覆盖面逐步扩大。完成道路改造、水环境建设、绿化美化等城建投资7亿元，荆沙大道、北京路、荆沙路、白云路、红门路、红星路及江津东路延伸线实现黑化升级，新建改建垃圾中转站22座、公厕40座，北京中路地下人防工程开工建设；绿化改造东门、小北门、新南门、塔桥北区及荆沙河等片区，新增绿地12万平方米；荆沙河、护城河、荆襄河清淤护岸、截污治污、水系连通和生态补水工程基本完成，城南污水处理厂建成投运，草市污水处理厂试运行，城区水环境大为改善。松滋、公安、石首城区及四湖流域10个乡镇污水治理项目完工，全市新增日污水处理能力21.6万吨。加强村镇规划建设和管理，争取村镇基础设施建设投入6000多万元。县市城区规模拓展，管理水平提高，继松滋、石首创建为省文明城市后，洪湖荣获中国优秀旅游城市和省卫生城市、园林城市称号，江陵、公安、监利卫生城市创建有序推进。

把加强精神文明和民主法治建设作为政府的重要职责，保持了社会和谐稳定。以庆祝新中国成立60周年为主题，深入开展群众性精神文明创建活动，公民思想道德素质和城乡文明程度进一步提高。“10.24”全国见义勇为英雄集体先进事迹感动中国，省委、省政府作出向英雄群体学习的决定，英雄群体代表在北京人民大会堂和部分大城市巡回报告，引起强烈反响，全市广大干部群众掀起学习英雄、热爱英雄、颂扬英雄的热潮，“人链”精神成为激励荆州人民奋发向上的宝贵财富。文化、广播影视、社会科学、文学艺术、新闻出版事业进一步繁荣。荆州电视台《垄上行》被评为新中国60年最有影响的60个广播电视栏目，有线电视整体转换和进村入户进展加快，市图书馆通过国家一级图书馆评估验收，第三次文物普查顺利完成，被省命名为关公文化之乡。建设乡镇综合文化站31个、农家书屋852个。全民健身活动深入开展，成功举办市第四届运动会，荣获全国群众体育先进单位称号。贯彻落实“五五”普法规划，稳步推进依法治理。认真汲取石首“6.17”事件教训，广泛开展矛盾大排查、纠纷大化解、民情大接访、问题大督办活动，切实加强大调解机制建设，办理法律援助案件1359件，设立社区法律援助接待室80个，妥善解决了一批重大疑难信访问题和矛盾纠纷。扎实推进平安荆州建设，深入开展“除五霸、抓五小、治毒害、端赌窝”等专项行动，社会治安形势进一步好转，荣获全国社会治安综合治理优秀市称号。组织开展生产安全、消防安全、食品药品安全等专项整治，安全事故起数、死亡人数等保持下降态势，连续四年受到省政府表彰。积极做好移民安置工作，四川汉源对口援建工程圆满完成。优抚安置、国防教育、国防动员、人民防空、预备役部队和民兵组织建设得到加强。妇女儿童和未成年人、老年人、残疾人合法权益得到保护，免费为280名贫困白内障患者实施复明手术，为114名贫困下肢残疾人安装假肢，为1000名贫困精神残疾人提供基本控制药物。

编制、统计、外事侨务、民族宗教、对台、气象、地震、档案、地方志、盐务、邮政和无线电管理等工作都取得了新进步。

在看到成绩的同时，我们也清醒地认识到，发展不快、发展不够、发展不优仍然是荆州最大的实际，经济社会发展中面临不少矛盾和困难，政府工作还存在一些问题和不足。主要表现在：一是受国际金融危机的严重冲击，化工、纺织等行业外需不足，全市外贸出口下降27.2%，经济回升基础不够牢固。二是工业经济总量不大，县域经济实力不强，民营经济活力不足，农民持续增收较为困难，改革创新和转变发展方式的任务艰巨。三是解决就业、社会保障、医疗卫生、困难群众生活等民生问题任务繁重，财政收支平衡难度增加。四是企业改制、征地拆迁、涉法涉诉等方面的社会矛盾较多，维稳压力较大。五是少数政府部门工作人员服务意识不强、工作作风不实、办事效率不高，基层、企业和群众仍有意见，政府自身建设有待进一步加强。对此，我们将高度重视，采取有效措施，认真加以解决。

2010年是全面完成“十一五”规划任务，为“十二五”发展打基础的关键之年。做好今年的政府工作，意义十分重大。

当前，加快发展的有利条件很多。一是世界经济逐步回暖，我国率先实现经济回升，发展环境好于上年。二是国家继续实施积极的财政政策和适度宽松的货币政策，大力推进“中部崛起”发展战略，特别是省委、省政府支持荆州在长江经济带开放开发和鄂西生态文化旅游圈建设中加快发展，在交通、港航、能源等基础设施、重点产业和城镇发展、文化旅游等方面给予项目和资金扶持，为我市增投资、上项目、调结构提供了重大机遇。三是随着“工业兴市”战略深入推进，历届市委、市政府“打基础、管长远”积蓄的能量持续释放，综合实力进一步增强，为加快发展步伐奠定了坚实基础。四是汉宜铁路、荆岳铁路、洪监高速、江南高速、引江济汉等一批重大项目开工和即将开工，为改善区位条件、优化投资环境提供了有力支撑。五是通过深入开展学习实践科学发展观活动，各级领导班子推动科学发展的能力进一步提升，全市广大干部群众求富思进、抢前争先的愿望强烈，为加快发展提供了强大的思想保证。在今年的工作中，我们将进一步振奋精神，抢抓机遇，埋头苦干，坚持保增长、转方式、调结构有机结合，坚持扩投资、促消费、稳出口有机结合，坚持抓发展、惠民生、保稳定有机结合，切实增强科学发展的自觉性、改革开放的坚定性、促进和谐的主动性，以更加积极进取的姿态、更加求真务实的作风，努力把改革发展稳定工作做得更好。今年重点抓好以下工作。

着力打造长江流域经济重镇。按照构筑大交通、培育大产业、建设大城市的发展思路，积极呼应“荆荆宜”城市群建设，精心编制“十二五”规划，加大产业带、城镇群和重点基础设施建设力度，把荆州建设成为湖北长江经济带的“钢腰”。突出项目策划，以港口、交通基础设施建设为先导，以推进新型工业化、城镇化为主题，以发挥长江水运优势、促进特色产业发展为重点，抓紧谋划一批带动能力强、支撑作用大的产业发展和基础设施项目。突出交通建设，重点打造荆州组合港，推进沿江公路、过境铁路、跨江通道、城市外环建设，加快建设水公铁联运、江海直达的长江中游综合交通枢纽。突出产业支撑，发展壮大以纺织、化工、机械汽配、轻工家电为特色的先进制造业，以电子信息、照明电器、生物医药、新材料等为特色的高新技术产业，以优质农产品生产和深加工为特色的现代农业，以文化旅游、金融保险、商贸物流为特色的现代服务业，努力建设长江中游现代产业带。突出城镇发展，强化规划引领、产业支撑和人口集聚，培育洪湖、监利、石首、松滋等节点城市，加快构建以中心城区为龙头、以沿江县城和重点城镇为依托的长江带城镇群。

（二）以“工业兴市”为主导，提升工业总量和竞争力。坚持“工业兴市”战略不动摇，着力打造百亿产业集群，扩大总量，优化结构，提高效益，确保规模以上工业增加值增长20%以上。

做实工业园区。继续实施“满园工程”，完善园区发展规划和产业定位，高标准推进园区水、电、路、环保等基础设施建设，强化招商引资和配套服务，提高园区建设和管理水平。荆州开发区重点抓好纺织印染循环工业园、电子工业园等“五园两中心”建设，提升产业承载能力和综合配套功能，积极争创国家级开发区，力争今年新引进过亿元项目10个以上，新投产、在建项目分别达到70个以上。县市区园区突出抓好项目引进和骨干培育，打造特色产业，推动差异化发展，力争通过2-3年的努力，每个县市区都建有规模过50亿元的特色园区。

做强县域工业。牢固树立“工业兴则县域兴、工业强则县域强”的理念，进一步强化“工业兴县”的发展举措，狠抓市场主体培育和软硬环境建设，积极争取省级专项资金扶持，推动企业向园区和城镇集中，促进县域工业大发展、大提高。高度重视企业家队伍建设，大力引进和培育企业经营管理人才。强化创业有功、致富光荣的观念，积极推进全民创业，鼓励支持回归创业，激活民间资本，努力提高自我发展能力。落实县市区主要领导抓工业发展的责任，组织开展县域工业发展量化考核，推动县域经济争先进位。

努力扩大投资规模。抢抓国家扩大内需的政策机遇，继续开展“项目建设年”活动，积极争取项目投资，特别是农业基础设施、保障性安居工程、教育、卫生等民生领域和自主创新、技术改造、节能环保等方面的投入，保持投资持续较快增长。按照策划一批、开工一批、储备一批的要求，充实完善项目库，保障重大项目建设的有序接替。实行“一个项目、一名领导、一套班子、一抓到底”的项目建设责任制，认真解决用地、融资等突出问题，加快项目建设进度。重点做好汉宜铁路、荆岳铁路、引江济汉等重大项目征地拆迁和协调服务，建成荆岳长江大桥和随岳南高速公路，尽早开工建设洪监高速、江南高速、郝穴至容城和盐卡疏港一级公路，以及荆州南500千伏输变电工程等项目，做好荆沙发电、松滋核电、长湖整治等项目前期工作。

努力扩大城乡消费。围绕建设江汉平原商贸物流枢纽城市，加强中心城区核心商圈建设，适应经济转型和消费结构升级的新变化，运用现代经营方式改造提升传统商贸、餐饮等服务业，积极引进摩尔城等国内外知名企业，增强集聚辐射功能。依托港口、公路、铁路等交通节点，高起点规划建设荆州火车站物流园、江陵危化品物流园、荆州港保税物流园等重点物流园区，促进生产性服务业有序发展。加强生产资料和农产品产地批发市场建设，支持两湖平原农产品交易物流中心建设成为具有贸易服务、市场营运、信息交流等功能的综合性平台。认真落实家电、汽车、摩托车、农机下乡等政策措施，扎实推进“万村千乡市场工程”，加快实施集村务、商务、服务为一体的村级综合服务社建设，不断激活农村消费。积极发展社区服务、社会养老等生活性服务业，培育文化体育、信息咨询、中介服务等新兴服务业，满足多层次、多样化的服务需求。推广连锁配送、电子商务等消费方式，提升现代服务业发展水平。整顿和规范农资、建材、成品油、烟草等市场秩序，努力营造便利、安全、放心的消费环境。同时，用足用活扶持外贸发展和出口退税等优惠政策，努力扩大农产品、机电产品出口，引导化工、纺织等行业开拓新兴市场，支持优势企业到境外开发资源、承包工程、投资办厂，力争外贸出口稳步回升。优化大通关服务，确保盐卡一类口岸通过验收。

积极发展现代农业。加强农业综合生产能力建设，稳定粮食生产，大力发展水产业，加快发展畜牧业，积极发展平原林业，抓好板块基地、小区片带和生产大县建设，提升优势产业规模化种养水平，力争水产放养面积增加8万亩、产量增加5万吨，生猪出栏增加30万头、家禽出笼增加1500万只。发展以种植、养殖、加工为主的庭院经济，多渠道增加农民收入。依托农产品资源优势，大力发展精深加工，狠抓监利福娃、洪湖水产、石首林业、公安中粮油料加工、荆州雨润、松滋白云边等十大加工园区及农产品加工项目建设，努力形成一批销售收入过100亿元的加工大县、过50亿元的加工园区、过20亿元的龙头企业和精品名牌产品，推动农产品生产大市向加工大市转变。积极推进江南农业高新技术园区建设，探索发展现代农业的新路子。扩大农业标准化生产，提高农产品质量安全水平。大力发展农民专业合作组织，完善农业科技公益性服务体系，强化重大动物疫病和农作物病虫害防控，提高农业专业化、市场化、社会化服务能力，全市农民专业合作社力争发展到700家以上，农机综合作业率提高5个百分点。

认真落实强农惠农政策。加大财政投入力度，引导社会资源投向农业农村，参与农村产业发展和公共设施建设。及时足额发放粮食直补、良种补贴、农机具购置补贴、农资综合补贴等资金，严格执行粮食最低收购价、油菜籽临时收储政策，保护和调动农民生产积极性。严格农民负担预算审批管理，规范农村一事一议和筹资筹劳，规范涉农收费，切实减轻农民负担。积极化解乡村债务，严格控制新增债务。

提升中心城区核心竞争力。围绕建设人水和谐城市目标，积极开展创建国家级卫生城市、国家级园林城市、国家级环保模范城市和省级文明城市“四城同创”活动，提升城市形象和品位。认真编制中心城区分区规划、控制性详规，严厉查处违规建设行为，切实维护规划的统一性和权威性。强化土地收购储备，增强城投公司融资功能，加快火车站片区、开发区综合服务区建设步伐，积极推进荆州古城人口疏散和街巷改造。实施中心城区道路升级改造、园林绿化、水环境保护等工程，完善城市道路、污水管网、生活垃圾无害化处理等设施，抓好城市出入口、沿河沿湖沿堤绿化景观带和城市游园建设，畅通城区水系，改善城市基础设施条件。进一步整治小街小巷、城乡结合部环境卫生、集贸市场占道经营和交通秩序，健全“门前三包”办法，建立长效管理机制，不断提高城市管理水平。加大城中村和危旧房改造力度，引导房地产业健康发展。

（六）以深化改革和管理创新为重点，增强经济社会发展活力。坚持以思想大解放促改革、促发展，创新体制机制，破解发展难题，推动经济社会又好又快发展。

加大增收节支力度。积极培植税源，做大财政蛋糕。加强税收征管和非税收入管理，严厉打击偷逃税行为，确保财政收入稳定增长。牢固树立过紧日子的思想，优化支出结构，加大对民生领域和社会事业、重点领域改革、重点项目建设的支持保障力度，从严控制非生产性支出。加强政府债务管理，健全政府举债、偿债机制，努力控制财政风险。完善部门预算管理、国库集中收付和政府采购等制度，严格执行收支两条线制度，提高财政资金的使用效益。

（七）以改善民生为着力点，协调发展各项事业。坚持优先保障和改善民生，促进公共资源配置更多地向民生领域倾斜，向解决热点、难点问题倾斜，确保社会和谐稳定。

加快发展社会事业。均衡发展城乡义务教育，扩大优质高中教育资源。大力发展职业教育，支持长江大学等高校健康发展。积极争取国家、省科技专项，加快科技信息服务平台建设和科技人才引进，促进科技进步。抓好县级医院、乡镇卫生院、农村卫生室和城市社区卫生服务中心建设，开展农村居民健康工程先进县和村镇示范卫生机构创建活动，完善城乡医疗卫生服务网络，切实做好甲型H1N1流感、血吸虫病、艾滋病等疫病防控工作。深化文化体制改革，抓好乡镇（街道）综合文化站、文化信息资源共享平台、农家书屋等建设，积极申报中国“三国文化之乡”，加强文物和非物质文化遗产保护，发展新闻出版、广播电视、文学艺术和社会科学事业，促进文化大发展、大繁荣。广泛开展全民健身活动，重视体育后备人才培养。健全计划生育利益导向机制，加强流动人口计生管理和出生人口性别比综合治理，稳定低生育水平。抓好工业、交通、建筑等重点领域节能和重点企业污染减排，加强城镇饮用水源保护和污水处理厂建设及运行监管，实施洪湖、四湖流域等重点区域生态修复，切实保护生态环境。强化基本农田保护，严格土地监管，促进节约集约利用。深入开展群众性精神文明创建活动，进一步提高市民素质和城乡文明程度。加强城乡社区建设，提高社会管理水平。落实优抚安置政策，争创新一轮省级双拥模范城。抓好第六次人口普查，加强外事侨务、民族宗教、对台工作，做好移民安置、气象、地震、档案、史志等工作，重视和支持老龄、残疾人事业发展。

三、加强政府自身建设，进一步提高政府执行力

切实推进依法行政。自觉增强法制意识，认真执行人大及其常委会的决议、决定，自觉接受人大法律监督和工作监督、政协民主监督和社会舆论监督，充分听取各民主党派、工商联和无党派人士的意见。扎实办理人大代表建议、批评、意见和政协委员提案，做到事事有着落，件件有回复。支持工会、共青团、妇联等群团组织的工作，发挥群团组织参与社会管理和公共服务的桥梁纽带作用。健全重大事项集体决策、专家咨询、社会公示与听证制度，提高决策科学化、民主化水平。完善政务公开和新闻发布制度，加强政府网站和电子政务建设，努力拓宽政务公开渠道，提高政府工作透明度。强化政府法制建设，抓好行政复议和应诉工作，严格落实行政执法责任制和过错责任追究制，规范行政行为。

切实优化发展环境。牢固树立亲商、安商、富商意识，强化服务基层、服务项目、服务企业意识，努力营造让投资者放心、安心、舒心的生产生活环境。深入推进行政服务网络建设，健全便民服务体系。深化行政审批制度“三集中三到位”改革，落实企业直通车服务、便民代办服务、首问负责等制度，优化审批流程，提高服务效率。认真清理行政事业性收费，规范收费行为。进一步量化、细化行政处罚裁量权，最大限度地减少执法随意性。落实优化经济环境问责办法，严肃查处影响企业生产和项目建设的违法行为，切实维护投资者的合法权益。

各位代表，为民办实事是政府义不容辞的职责。今年，市政府将进一步加大投入，继续办理十件实事，用实事顺应民愿，以实效凝聚民心。（1）城镇就业新增7万人，农村劳动力转移新增就业3.5万人、 培训3.4万人；将全市10%农业人口纳入社会养老保险体系。（2）为农村4.5万名义务教育阶段经济困难寄宿生补助生活费，为城市义务教育阶段低保家庭学生免费提供教科书，向3.7万名中职学生发放助学金。（3）提高财政对城乡居民医保的补助标准，扩大城乡居民基本医疗保险门诊统筹覆盖面，免费救治晚血病人2600人。（4）新建通村公路1200公里、农村候车亭180个。（5）新建或改造农家店300家，解决农村38.87万人的饮水安全问题，补贴农户购买小型耕整机3000台。（6）建设30个乡镇文化站、500家农家书屋，维修改造荆州市群众艺术馆。（7）建立9个标准化职工法律援助中心，为困难职工和农民工免费提供法律援助，办理法律援助案件1100件以上。（8）开展中心城区范家渊公园、荆襄河公园建设和太湖港（古城区段）景观改造，黑化升级荆东路、荆北路、黄金堂路、南湖路、园林路、江汉路、豉湖路。（9）中心城区新建廉租住房400套，发放住房租赁补贴1万户，启动6万平方米经济适用住房建设。（10）免费为200名贫困白内障患者实施复明手术，为100名贫困下肢残疾人安装假肢，为1000名贫困精神病患者提供基本控制药物，启动6岁以下贫困残疾儿童抢救性康复项目。
